# Antidiuretic hormone release associated with increased intracranial pressure independent of plasma osmolality

**DOI:** 10.1002/brb3.1005

**Published:** 2018-05-23

**Authors:** William J. Keller, Elda Mullaj

**Affiliations:** ^1^ Saint James School of Medicine—Anguilla The Valley Anguilla, British West Indies

**Keywords:** ADH secretion, antidiuretic hormone secretion, arginine vasopressin, hyponatremia, increased intracranial pressure

## Abstract

**Objective:**

Introduce and evaluate a new model which explains the release of brain antidiuretic hormone (ADH) independent of plasma osmolality.

**Methods:**

Systematic review and critical analysis of the professional literature.

**Results:**

Primary electronic database searches using key terms revealed 57,432 hits. Secondary searches with application of specific inclusion and exclusion criteria and manual inspection for completeness reduced the total number of studies to fourteen (*N* = 14). Twelve (*N* = 12) studies investigated human subjects in the hospital settings, and two (*N* = 2) studies investigated animals (rhesus monkeys and dog) under invasive experimental conditions. All fourteen studies included direct or indirect indicators of intracranial pressure (ICP), measurements of plasma ADH, and plasma osmolality or urine osmolality. Findings, in brief, reveal a stable and positive association between increased intracranial pressure (ICP) and increased ADH release, in patients with low or normal blood osmolality. Findings are reliable and reproducible across human and animal populations.

**Conclusions:**

Findings support the proposed model, which explains increase secretion of brain ADH when plasma osmolality is low or within normal limits. Mechanical pressures exerted on hypothalamic nuclei, especially paraventricular and supra‐optic nuclei, as a consequence of increased intracranial pressure, produce release of ADH, independent of plasma osmolality. The mechanical pressure model explains release of ADH previously unexplained by traditional plasma osmolality models. Findings have important clinical implications for the medical and surgical management of patients.

## 1 INTRODUCTION

This study systematically reviews the professional literature and evaluates a new model within the matrix of past and current scientific knowledge. The proposed model offers a new conceptual matrix which explains release of antidiuretic hormone (ADH), independent of blood osmolality. Findings are interpreted within the context and applications for the practicing physician, especially those who manage patients presenting with disturbance of electrolyte imbalances, for example hyponatremia, when blood osmolality values are low or within normal limits.

The existing body of experimental and clinical research literature fails to provide an adequate or unifying theory as to why increased intracranial pressure (ICP) is associated with increased secretion of ADH, when plasma osmolality is low or within normal limits. Our proposed model explains how increased ICP produces an increase in ADH release, offers a mechanism of action independent of plasma osmolality, and offers a theory based explanation as to why clinical conditions such as hyponatremia can develop in the presence of normal plasma osmolality.

Traditional models emphasize plasma osmolality as primary in initiating and maintaining ADH release. As plasma osmolality increases ADH is released, returning plasma osmolality to normal values. Over the past 70 years, investigators repeatedly report increases in ADH secretion when plasma osmolality is high. These findings are explained within the traditional osmolality models and are well understood. However, in the presence of increased ICP, there is frequently a subgroup of patients who evidence increased secretion of ADH in the presence of low or normal plasma osmolality. Investigators periodically have offered explanations to account for these findings; however, none of the models venture far from the traditional osmolality model.

For example, nearly fifty years ago, Wise reported enlargement of the third ventricle is associated with increased ADH release. He attributes the increased ADH release to disruption of hypothalamic osmo‐receptors (Wise, 1968).

Gaufin, Swowsky, and Goodman, working with rhesus monkeys and investigating nonosmotic triggers potentially responsible for ADH release, reported the magnitude of ADH release is directly associated with the magnitude of intracranial pressures. As intracranial pressures increase, ADH release increases. Gaufin, Swowsky, and Goodman are among the first to offer data implicating nonosmotic triggers and offer the foundation upon which the proposed model is based (Gaufin, Skowsky, & Goodman, 1977).

Current prevailing models explain the release of ADH as the result of increased osmotic pressure, produced by increased plasma osmolality. These models correctly and adequately explain the relationship between ADH release and plasma osmolality for healthy individuals and for most patients presenting with common electrolyte imbalances, such as hyponatremia. However, these models fail to adequately explain increased ADH release when plasma osmolality is low or within normal limits.

Our proposed model in brief states, mechanical pressure exerted on hypothalamic nuclei, specifically paraventricular and supra‐optic nuclei, mechanically and directly stimulates firing of these neurons, resulting in the release of ADH. The mechanism of stimulation is completely independent of the typical and traditionally accepted mechanism of stimulation by way of increased blood osmolality and brain osmo‐receptors.

Specific questions tested by this study are:

1. Does antidiuretic hormone secretion increase under conditions of increased intracranial pressure?
2. Does antidiuretic hormone secretion increase under conditions of normal or low plasma osmolality?
3. Does antidiuretic hormone secretion increase under conditions of normal or low plasma osmolality and increased intracranial pressure?
4. Does antidiuretic hormone secretion change as a function of increased and decreased intracranial pressure, within the same subject?

## 2 METHODS

### 2.1 Research design

A systematic review of the professional medical literature was used to summarize existing knowledge, test specific research questions, and evaluate the proposed model.

### 2.2 Electronic databases

The scientific and medical literature was systematically searched electronically using the following four electronic databases: PubMed, Google Scholar, Embase, and OVID.

### 2.3 Specific key search terms

ADH secretion, antidiuretic hormone secretion, arginine vasopressin, hyponatremia, increased intracranial pressure.

### 2.4 Search strategy

An initial search was conducted using the specific key search terms. A secondary search was then conducted with refined and specific inclusion and exclusion criteria. Results from the secondary search were independently reviewed by two investigators (EM,WK) for final selection. Reference lists in each article identified by the secondary search were examined manually and relevant articles identified in the reference lists and not identified by the electronic search were added to the final study list. Studies identified as meeting all inclusion and exclusion criteria and containing all essential information; for example, measurements of ICP, ADH, plasma osmolality, or urine osmolality were placed on the final study list and independently reviewed by two investigators (EM, WK) for completeness. Only studies meeting all inclusion and exclusion criteria and independently confirmed by both investigators were retained and subjected to statistical analysis and summary.

### 2.5 Inclusion criteria

Studies appearing in the medical literature between the years 1954 and 2015 were included. Studies must have reported data containing measurements of ICP, ADH, plasma osmolality, or urine osmolality. It was not necessary for the study to specifically investigate the relationship between ICP, ADH, plasma osmolality, or urine osmolality, only that these measurements appear in the study and could be extracted. Studies were searched and extracted independent of subject's age, sex, or underlying patient pathology. Studies were searched and extracted independent of the language of the written article. Relevant non‐English articles were translated into English using Google Translate. Human and animal studies were included in all searches and study extractions. Unpublished studies presented at professional meetings, which appear as published abstracts, and published articles appearing in the professional medical literature between the years 1954 and 2015 were included in all searches and study extractions.

### 2.6 Exclusion criteria

Studies that did not report measurements of ICP, ADH, plasma osmolality, or urine osmolality were excluded.

### 2.7 Data extraction

Studies meeting inclusion and exclusion criteria were compiled. The following data were extracted from each study: article title, article authors, year of publication, subject characteristics for example age, sex, and identified pathology if any, number of subjects in each study, number of subjects evidencing increased release of ADH, number of subjects demonstrating increased release of ADH when plasma osmolality were low or within normal limits, measurements of ICP, measurements of ADH, measurements of plasma osmolality, and measurements of urine osmolality.

### 2.8 Data summary and statistical analysis

Data extracted from each study were summarized into two tables. Subject characteristics were summarized by descriptive statistics for all studies appearing in the summary tables. Studies reporting percentage measurements or raw numbers for primary measurements of interest, ICP, ADH, plasma osmolality, and urine osmolality were converted into standard measurement units to facilitate comparisons across studies. Group values, when reported as maximum and minimums, ranges, or medians and when the requisite additional information could be extracted from the original article, were converted into means and standard deviations, for the purpose of parametric statistical analyses. Descriptive summary statistics were computed for each primary measurement of interest, ICP, ADH, and plasma osmolality. A priori group and subgroup statistical analyses were completed to test specific research hypothesis and test the proposed new model.

## 3 RESULTS

**Figure 1:**
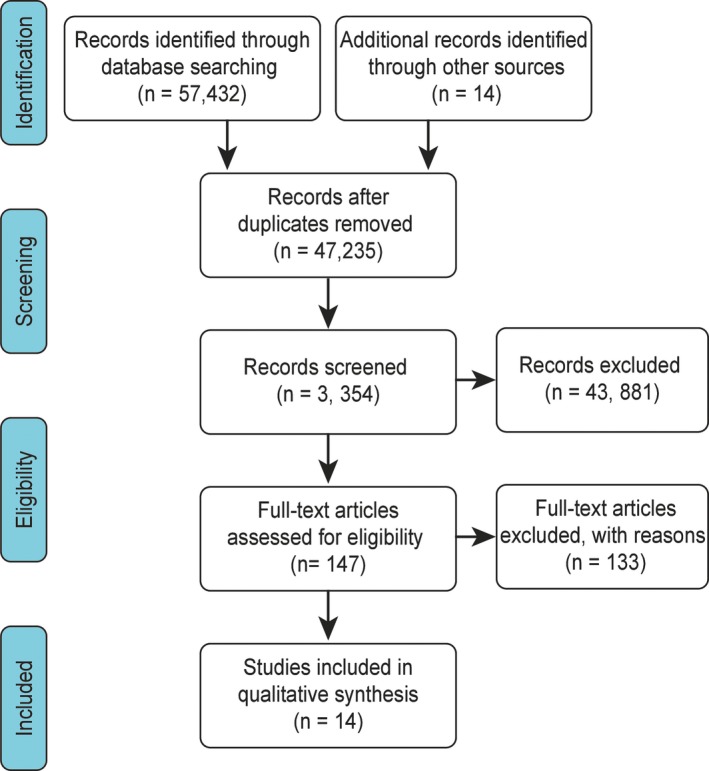
Preferred Reporting Items for Systematic Reviews and Meta‐Analysis (PRISMA) (Moher, Liberati, Tetzlaff, & Altman, 2009)

The primary electronic search conducted using the key terms resulted in 57,432 hits. The secondary electronic search, applying specific inclusion and exclusion criteria, reduced the count to 132 hits. Manual inspection of the reference lists in each of the 132 articles, identified in the secondary electronic search, resulted in the identification of 15 additional studies. The final list of 147 studies was manually inspected for completeness and data extraction (Figure 1).

Fourteen (*N* = 14) studies met all criteria and were subjected to statistical analysis. The studies (*N* = 14) represent investigations completed over the course of 42 years (1968–2010). Twelve (*N* = 12) studies investigated human subjects in the hospital setting (Brown & MacDonald, 1981; Cotton et al., 1993; Mather, Ang, & Jenkins, 1981; Miura, Takagi, Matsukado, & Ushio, 1989; Sazbon & Groswasser, 1990; Sørensen, Gjerris, & Hammer, 1985a, 1985b; Vingerhoets & De Tribolet, 1988; Widmayer, Browning, Gopinath, Robertson, & Baskin, 2010; Wijdicks et al., 1985; Wise, 1968; Yoshino, Yoshimi, Taniguchi, Nakamura, & Ikeda, 1999) and two (*N* = 2) studies investigated animals (rhesus monkeys and dog) under invasive experimental conditions (Gaufin et al., 1977; Shiel, Pinilla, & Mooney, 2009). All fourteen (*N* = 14) studies included direct or indirect indicators of increased intracranial pressure, for example direct measurement, computerized tomography, and clinical assessment. All studies included space occupying cerebral lesions, for example aneurysms of the anterior communicating artery, intracerebral tumor, cerebral hemorrhage, controlled balloon inflation, cerebral edema and affecting directly or indirectly the hypothalamic nuclei, particularly paraventricular and supra‐optic nuclei. Studies included measurements taken immediately upon initial onset of increased intracranial pressure, as well as measurements taken several months following sustained, increased intracranial pressure.

Table 1 summarizes the findings from twelve (*N* = 12) human subject studies.

**Table 1:** Human studies

Authors	Journal	Year	Population	Pathology and No. of patients	Intracranial pressure (mmHg)	Plasma ADH (pg/ml)	Plasma osmolality (mOsm/kg water)	Urine osmolality (mOsm/kg water)	Notes
1. Wise	Journal of Neurosurgery	1968	Adult	Hydrocephalus, blocked shunts*N* = 2			PretreatmentPt. 1 Not reportedPt. 2 255 mOsm/kg	PretreatmentPt. 1 828 mOsm/kgPt. 2 631 mOsm/kg	Clearing the shunts resolves the hydrocephalus and improves plasma and urine osmolality.
							PosttreatmentPt. 1 Not reportedPt. 2 289 mOsm/kg	PosttreatmentPt. 1 Not reportedPt. 2 116 mOsm/kg	
2. Brown and MacDonald	Pediatric Research	1981	Pediatric	Reye's syndrome*N* = 9	PretreatmentIncreased ICP, “… first 72 hr ICP monitoring…”.	PretreatmentRange = 4.2–10.0 uIU/mlRange = 1.7–4.1 pg/ml			Original paper reports plasma ADH values in uIU/ml. These values have been converted to pg/ml for easy, cross study comparisons in this table.
					PosttreatmentNormal ICP, “… after recovery…”.	PosttreatmentRange = 0.5–2.2 ulU/mlRange = 0.21–1.98 pg/ml			
3. Mather, Ang, and Jenkins	Journal of Neurology, Neurosurgery and Psychiatry	1981	Adult	Space occupying cerebral lesion: Subarachnoid hemorrhages—source: anterior communicating artery*N* = 6		Mean = 5.51 pmol/L*SD* = 2.17 pmol/L	Mean = 278 mOsm/kg*SD* = 14 mOsm/kg		Original paper reports plasma ADH values in pmol/L. These values have been converted to pg/ml for easy, cross study comparisons in this table.Pts 1,2,3,4,6,10.
						Mean = 5.96 pg/ml*SD* = 2.35 pg/ml			
4. Wijdicks, Vermeulen, Tenhaaf, Hijdra, Bakker, and van Gijn	Annals of Neurology	1985	Adult	Healthy controls*N* = 7		Mean = 0.65 pg/ml*SD* = 0.16 pg/mlRange = 0.5–0.9 pg/ml			
				Space occupying cerebral lesion:Subarachnoid hemorrhage‐source: ruptured aneurysm*N* = 14		PretreatmentMean = 4.9 pg/ml*SD* = 5.7 pg/mlRange = 0.4–19 pg/ml			Authors suggest the initial, pretreatment; rise in ADH levels may be related to a sudden increase in ICP.
						PosttreatmentMean = 1.25 pg/ml*SD* = 0.78 pg/mlRange = 0.1–2.5 pg/ml			
5. Sorensen, Gjerris, and Hammer	Journal of Neurology, Neurosurgery and Psychiatry	1985	Adult	Patient controlsNo CNS disordersNo endocrine d/o*N* = 52		Mean = 3.1 pg/ml*SD* = 1.4 pg/ml*SEM* = 0.2 pg/ml	Mean = 286 mOsm/kg*SD* = 7.2 mOsm/kg*SEM* = 1 mOsm/kg		Authors suggest high ICP may have been the cause of increased ADH in these patients.
				Space occupyingCNS lesions*N*=62	<18 mmHg	Mean = 3.6 pg/ml*SD* = 1.1 pg/ml*SEM* = 0.1 pg/ml	Mean = 280 mOsm/kg*SD* = 6.7 mOsm/kg*SEM* = 0.85 mOsm/kg		
				HPH* *n* = 6ICT* *n* = 14BIH* *n* = 18ICH* *n* = 9IS* *n* = 10CCT* *n* = 5		Mean = 2.9 *SEM* = 0.4Mean = 4.9 *SEM* = 1.1Mean = 2.8 *SEM* = 0.4Mean = 5.0 *SEM* = 1.1Mean = 2.7 *SEM* = 0.8Mean = 3.4 *SEM* = 1.0	Mean = 279 *SEM* = 4Mean = 285 *SEM* = 2Mean = 284 *SEM* = 1Mean = 280 *SEM* = 8Mean = 288 *SEM* = 2Mean = 269 *SEM* = 10		Hydrocephalus*Tumor*Intracranial HTN*Hemorrhage*Ischemic stroke*Cerebral trauma
				Non‐ space occupying CNS lesions*N*=38		Mean = 1.96 pg/ml*SD* = 1.05 pg/ml*SEM* = 0.17 pg/ml	Mean = 285 mOsm/kg*SD* = 1.0 mOsm/kg*SEM* = 0.16 mOsm/kg		
				MS* *n* = 8BGD* *n* = 5PDD* *n* = 25		Mean = 3.0 *SEM* = 0.4Mean = 2.0 *SEM* = 0.3Mean = 0.9 *SEM* = 0.1	Mean = 285 *SEM* = 3Mean = 284 *SEM* = 2Mean = 286 *SEM* = 1		Multiple Sclerosis*Basal Ganglia D/O*Primary dementia
				Psychiatric pt.controls*N* = 62ED* *n* = 32NED* *n* = 14Mania* *n* = 7Schiz* *n* = 9		Mean = 2.55 pg/ml*SD* = 0.7 pg/ml*SEM* = 0.01 pg/ml	Mean = 287 mOsm/kg*SD* = 0.95 mOsm/kg*SEM* = 0.12 mOsm/kg		*Endo‐depression*Non‐Endo‐depression*Mania*Schizophrenia
						Mean = 2.6 *SEM* = 0.3Mean = 2.4 *SEM* = 0.3Mean = 2.6 *SEM* = 0.5Mean = 2.6 *SEM* = 0.4	Mean = 288 *SEM*=1Mean = 288 *SEM*=2Mean = 286 *SEM*=3Mean = 287 *SEM*=2		
6. Sorensen, Gjerris, and Hammer	Acta Neuro‐chirurgica	1985	Adult	Hydrocephalus*N* = 8	Low pressureMean = 7 mmHg*SD* = 2.5 mmHg*SEM* = 1 mmHgRange = 4–10 mmHg	Mean = 2.4 pg/ml*SD* = 1.2 pg/ml*SEM* = 0.4 pg/mlRange = 0.9–4.9 pg/ml			ADH levels exceeded 50% increase in 6 of 8 patients from Low ICP to High ICP conditions.
					High pressureMean = 27 mmHg*SD* = 9.6 mmHg*SEM* = 2 mmHgRange = 22–33 mmHg	Mean = 4.2 pg/ml*SD* = 2.2 pg/ml*SEM* = 0.8 pg/mlRange = 2.3–9.6 pg/ml			
7. Vingerhoets and de Tribolet	Acta Neuro‐chirurgica	1988	Adult	Severe headtrauma; within 72 hr after admission*N* = 3		Mean = 2.6 pg/ml*SD* = 0.17 pg/ml*SEM* = 0.09 pg/mlRange = 2.4–2.7 pg/ml	Mean = 271 mOsm/kg*SD* = 12.2 mOsm/kg*SEM* = 7.0 mOsm/kgRange = 257–280 mOsm/kg	Mean = 948 mOsm/kg*SD* = 190 mOsm/kg*SEM* = 109 mOsm/kgRange = 729–1,060 mOsm/kg	Pts 1, 2, 3.
8. Miura, Takagi, Matsukado, and Ushio	Neurologia Medico‐Chirugica	1989	Adult	Space occupyingCNS lesions*N* = 20		Mean = 22.48 pg/ml*SD* = 8.48 pg/ml*SEM* = 1.89 pg/ml			Original Groups B+D+E.
				Group B (elevated ADH in plasma with normal Na+*n* = 4		Mean = 32.08 pg/ml*SD* = 18.24 pg/ml*SEM* = 9.12 pg/ml			
				Group D (elevated ADH in plasma and CSF with normal Na+*n* = 8		Mean = 19.41 pg/ml*SD* = 10.24 pg/ml*SEM* = 3.63 pg/ml			
				Group E (elevated ADH in plasma with low Na+*n* = 8		Mean = 15.96 pg/ml*SD* = 10.95 pg/ml*SEM* = 3.88 pg/ml			
9. Sazbon and Groswasser	Journal Neurosurgery	1990	Adult	Severe blunt head trauma, e.g. motor vehicle accidents resulting in hypothalamic damage*N* = 16		“…abnormal ADH secretion…”			Prolonged coma lasting >30 days.Glasgow Coma Scale <7.
10. Cotton, Donald, Schoeman, van Zyl, Aalbers, and Lombard	Child's Nervous System	1993	Pediatric	Tuberculous meningitis.*N* = 24	Mean = 23.5 mmHg*SD* = 11.3 mmHg*SEM* = 2.3 mmHgMedian = 19 mmHgRange = 10–51 mmHg	Range = 0.3–24.5 pg/ml*N* = 24	Range = 246–289 mOsm/kg*N* = 27	Range = 168–655mOsm/kg*N* = 21	Measurements restricted to pts who had ICP, ADH, and urine collected on the same day.
				Group with SIADH*n* = 14	Mean = 26.5 mmHg*SD* = 12.3 mmHgMedian = 22.5 mmHgRange = 8.4–51 mmHg	Median = 4.5 pg/mlRange = 2.2– 24.5 pg/ml*n* = 13	Median = 262 mOsm/kgRange = 246–269 mOsm/kg*n* = 15	Median = 398 mOsm/kgRange = 168–667 mOsm/kg*n* = 14	
				Group without SIADH*n* = 12	Mean = 19.0 mmHg*SD* = 8.7 mmHgMedian = 18 mmHgRange = 10–41 mmHg	Median = 3.2 pg/mlRange = 0.3–17.2 pg/ml*n* = 11	Median = 278 mOms/kgRange = 264–289 mOsm/kg*n* = 12	Median = 484 mOsm/kgRange = 235–655 mOsm/kg*n* = 7	
11. Yoshino, Yoshimi, Taniguchi, Nakamura, and Ikeda	Internal Medicine	1999	Adult	Idiopathic Normal Pressure Hydrocephalus*N* = 1		Pretreatment14.0 pg/ml	Pretreatment256 mOsm/kg	Pretreatment367 mOsm/kg	Ventricular ‐peritoneal shunting returns low serum Na+ to normal range.
						Posttreatment3.2 pg/ml	PosttreatmentValue not reported	PosttreatmentValue not reported	
12. Widmayer, Browning, Gopinath, Robertson, and Boskin	Neurological Research	2010	Adult	Controls; No head trauma or injury*N* = 12	“…Normal ICP…”.Values not reported	Mean = 0.4 pg/ml			
				Head injury*N* = 11	Max pressureMean = 40 mmHg*SD* = 15.9 mmHgMedian = 43 mmHgRange = 14–67 mmHg	Mean = 4.1 pg/ml			Glasgow Coma ScaleMean = 7.5*SD* = 3.5Range = 3–14

*Note*. ADH, Antidiuretic hormone; ICP, intracranial pressure.

Table 1. Reference values.

Normal ICP = 7–15 mmHg, Emedicine Medscape (Gupta, 2015).

Normal ADH values in plasma = 0.2–1.7 pg/ml, Merck Manual Professional Edition (Wians, 2015).

Normal plasma osmolality = 275–295 mOsm/kg water, Merck Manual Professional Edition (Wians, 2015).

Normal urine osmolality = 500–800 mOsm/kg water, Emedicine Medscape (Wilczynski, 2014).

Empty cells indicate data were not reported or could not be extracted from the original study.

Table 2 summarizes the findings from two (*N* = 2) animal studies.

**Table 2:** Animal studies

Authors	Journal	Year	Population	No. of Animals	Intracranial pressure (mmHg)	Urinary ADH (μU/15 min)	Plasma osmolality (mOsm/kg water)	Urine Osmolality (mOsm/kg water)
13. Gaufin, Skowsky, and Goodman	Journal Neurosurgery	1977	Rhesus monkeys	Normal and healthy; experimental subdural balloon expansion*N* = 4	ControlsMean = <10 mmHg*n* = 4	Mean = 783 μU/15 min*SD* = 250 μU/15 min*SEM* = 125 μU/15 min		
					Non‐lethal ICP conditionMean = 65 mmHg*n* = 4	Mean = 3,433 μU/15 min*SD* = 528 μU/15 min*SEM* = 269 μU/15 min		
					Lethal ICP conditionMean = 100 mmHg*n* = 4	Mean = 4,339 μU/15 min*SD* = 3,774 μU/15 min*SEM* = 1,887 μU/15 min		
14. Shiel, Pinilla, and Mooney	Journal of the American Animal Hospital Association	2009	Dog	Severe hydrocephalus*N* = 1			On presentation233 mOsm/kg	On presentation958 mOsm/kg
							1 week later281 mOsm/kg	
							4 months later275 mOsm/kg	4 months later924 mOsm/kg

*Note*. ADH, Antidiuretic hormone.

Empty cells indicate data were not reported or could not be extracted from the original study.

Data from six primary studies were extracted, reorganized, and reinterpreted within the matrix of the proposed model and presented here. The studies were selected as representative of the professional literature reviewed between the years 1954 and 2015, utilizing multiple research designs, for example repeated measures with subjects as their own controls and between groups designs, and a broad range of subject populations, for example healthy individuals, hospitalized patients, children, adults, and animals.

Specific questions tested and answered:

1. Does anti‐diuretic hormone secretion increase under conditions of increased intracranial pressure? Yes.

**Figure 2:**
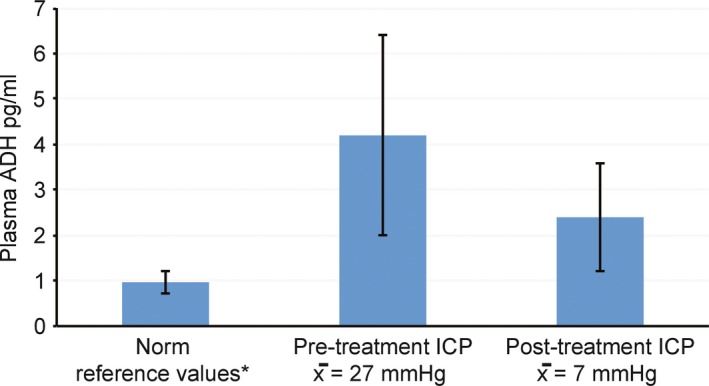
Plasma antidiuretic hormone as a function of intracranial pressure in eight adults with hydrocephalus. Mean ± *SD*. Original data extracted and reanalyzed from Sorensen, Gjeeris, and Hammer (Sørensen et al., 1985a). See Table 1, Study 6. *Norm reference values from Merck Manual Professional Edition (Wians, 2015)

Data originally reported by Sorensen, Gjerris, and Hammer report increased ADH secretion associated with increased ICP in eight (*N* = 8) adult patients, presenting with hydrocephalus. The study employed a repeated measure within group research design. Plasma ADH was measured and assessed under pretreatment (high) ICP and posttreatment (normal) ICP conditions (Sørensen et al., 1985a). Data from the original study have been extracted, reorganized, and reanalyzed here. See Table 1 (Study 6) and Figure 2.

Under the pretreatment, high ICP conditions (*M* = 27.0 mmHg, *SD* = 9.6) patients revealed abnormally high levels of ADH (*M* = 4.2 pg/ml, *SD* = 2.2). Under the posttreatment, normal ICP conditions (*M* = 7 mmHg, *SD* = 2.5) patients' ADH levels returned to normal (*M* = 2.4 pg/ml, *SD* = 1.2). Statistical analysis reveals a significant difference in ADH levels between the pretreatment (high) ICP and posttreatment (normal) ICP conditions, *t*(7) = 3.37, *p* < 0.01, with plasma ADH levels highest during the pretreatment (high) ICP condition and returning to normal limits during the posttreatment (normal) ICP condition. Findings are consistent with the proposed model. ADH secretion increases under conditions of increased ICP.

**Figure 3:**
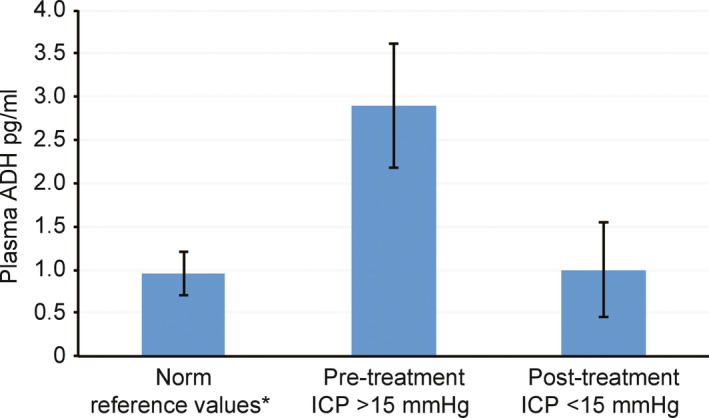
Plasma antidiuretic hormone as a function of intracranial pressure in nine children with acute encephalitis (Reye's syndrome) before and after treatment. Mean ± *SD*. Original data extracted and reanalyzed from Brown and MacDonald (Brown & MacDonald, 1981). See Table 1, Study 2. *Norm reference values from Merck Manual Professional Edition (Wians, 2015)

Data originally reported by Brown and MacDonald examines increased ADH secretion associated with increased ICP in nine (*N* = 9) pediatric patients, presenting with Reye's syndrome. The study employed a repeated measure within group design. Plasma ADH was measured pretreatment high ICP conditions and posttreatment normal ICP conditions (Brown & MacDonald, 1981). Data from the original study have been extracted, reorganized, and reanalyzed here. See Table 1 (Study 2) and Figure 3.

Under the pretreatment, high ICP condition (ICP > 15 mmHg) patients revealed abnormally high levels of ADH (*M* = 2.9 pg/ml, *SD* = 0.72). Under the posttreatment, normal ICP condition (ICP < 15 mmHg) patients' ADH levels returned to normal (*M* = 1.0 pg/ml, *SD* = 0.55). Statistical analysis reveals a significant difference in ADH levels between the pretreatment high ICP condition and posttreatment normal ICP condition, *t*(16) = 6.29, *p* < 0.0001, with plasma ADH levels highest during the pretreatment high ICP condition and within normal limits during the posttreatment normal ICP condition. Findings are consistent with the proposed model. ADH secretion increases under conditions of increased ICP.

1. Does antidiuretic hormone secretion increase under conditions of normal or low plasma osmolality? Yes.

**Figure 4:**
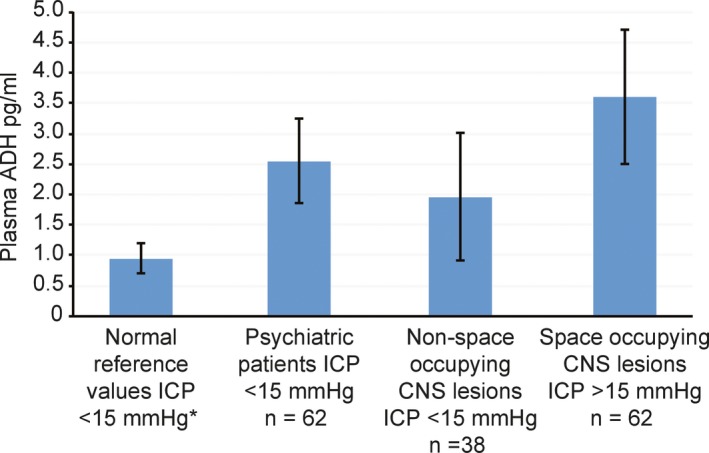
Plasma antidiuretic hormone as a function of intracranial pressure, across three patient groups, when plasma osmolality remains within normal limits in all patient groups. Mean ± *SD*. Data extracted and reanalyzed from Sorensen, Gjerris, and Hammer (Sørensen et al., 1985b). See Table 1, Study 5. *Norm reference values from Merck Manual Professional Edition (Wians, 2015)

Data originally reported by Sorensen, Gjerris, and Hammer examine the relationship between plasma ADH and plasma osmolality in adult patients, with and without increased ICP. The study employed a between independent group research design. Plasma ADH and plasma osmolality were measured in 243 patients with assorted neurological disorders, psychiatric disorders, and patient controls (Sørensen et al., 1985b). Data from the original study have been extracted, reorganized, and reanalyzed here. See Table 1 (Study 5) and Figure 4.

The original data were reorganized into four independent groups, patient controls with no central nervous system or endocrine disorders (*n* = 52), psychiatric patient controls (*n* = 62), patients with no space occupying brain lesions (*n* = 38), and patients with space occupying brain lesions (*n* = 62). All patients included in the space occupying brain lesion group revealed increased ICP >18 mmHg, at the time plasma ADH sample were taken. The patient control group, psychiatric patient group, and nonspace occupying brain lesion group revealed normal ICP <15 mmHg, at the time plasma ADH samples were taken. Twenty‐nine (*n* = 29) patients, presenting with normal pressure hydrocephalus and ICP <12 mmHg were excluded from the analysis, reducing the total number of patients and controls from 243 in the original study to 214. The normal pressure hydrocephalus patients were excluded from our analysis due to our inability to establish with certainly the underlying cause producing the NPH, for example nonspace occupying lesion or space occupying lesion. Plasma osmolality remained within normal limits for all four groups, at the time plasma ADH was sampled.

Statistical analyses reveal the space occupying brain lesion group releases significantly higher amounts of plasma ADH when compared to normal reference values *t*(122) = 18.49, *p* < 0.001, psychiatric patient group *t*(122) = 10.44, *p* < 0.0001, or nonspace occupying brain lesion group *t*(98) = 7.36, *p* < 0.001. Findings are consistent with the proposed model. ADH secretion increases under conditions of increased ICP when plasma osmolality remains within normal limits.

Data originally reported by Yoshino, Yoshimi, Taniguchi, Nakamura, and Ikeda, a case study report of idiopathic normal pressure hydrocephalus, revealed high ADH secretion during a condition of increased ICP (blocked ventricular shunt), while plasma osmolality remained low and below normal limits (256 mOsm/kg) (Yoshino et al., 1999). See Table 1 (Study 11).

During the pretreatment (blocked shunt) condition, the patient's plasma ADH was significantly elevated (14.0 pg/ml) and above normal reference values. Once the shunt was cleared (posttreatment condition), plasma ADH levels returned to normal (3.2 pg/ml) and plasma osmolality continued to remain within normal limits. Findings are consistent with the proposed model. ADH secretion increases under conditions of increased ICP when plasma osmolality remains low or within normal limits.

1. Does antidiuretic hormone secretion increase under conditions of normal or low plasma osmolality AND increased intracranial pressure? Yes.

Data originally reported by Cotton, Donald, Schoeman, Van Zyl, Aalbers, and Lombard examined the relationship between plasma ADH under conditions of normal or low plasma osmolality and increased intracranial pressure in thirty‐one (*N* = 31) pediatric patients presenting for hospital treatment with tuberculous meningitis. The study employed a between independent group research design (Cotton et al., 1993). Data from the original study have been extracted, reorganized, and reanalyzed here. See Table 1 (Study 10).

Raw data from twenty‐four (*N* = 24) of the original thirty‐one (*N* = 31) pediatric patients could be extracted from the original article, with all of the necessary measurements required, ICP, plasma ADH, and plasma osmolality. The 24 patients were divided into the two original independent groups, patients presenting with the syndrome of inappropriate antidiuretic hormone secretion (SIADH) (*n* = 14) and patients presenting without the SIADH (*n* = 10). Statistical analyses reveal no statistical difference between the two groups with respect to ICP *t*(22) = 1.65, *p* = 0.11 or plasma ADH *t*(22) = 0.36, *p* = 0.72. However, statistical analyses reveal significantly elevated levels of ICP and plasma ADH appearing in both groups when compared to normal reference values. Plasma osmolality remained within normal limits or low in both groups. Findings are consistent with the proposed model. ADH secretion increases with increased ICP when plasma osmolality is low or within normal limits.

1. Does antidiuretic hormone secretion change as a function of increased and decreased intracranial pressure within the same subject? Yes.

**Figure 5:**
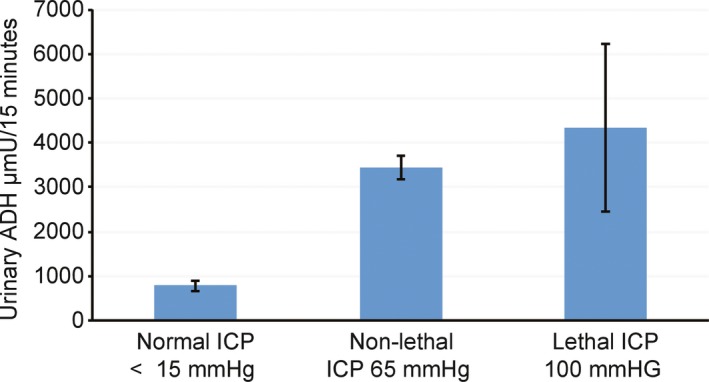
Changes in urinary antidiuretic hormone as a function of normal, nonlethal, and lethal levels of intracranial pressures in four Rhesus monkeys. Mean ± *SEM*. Each animal served as its own control. Original data extracted ad reanalyzed from Gaufin, Showsky, and Goodman (Gaufin et al., 1977). See Table 2, Study 13

Data originally reported by Gaufin, Skowsky, and Goodman examined the relationship between ICP and urinary ADH in four (*N* = 4) rhesus monkeys, under conditions of subdural, mass balloon expansion, and intravenous administration of hypertonic and hypotonic solutions. The study employed an experimental within subject repeated measures research design (Gaufin et al., 1977). Data from the original study have been extracted, reorganized, and reanalyzed here. See Table 1, (Study 13) and Figure 5.

Antidiuretic hormone secretion systematically increased with increased ICP. Statistical analyses revealed a statistically significant increase in ADH secretion when values are compared between the normal ICP condition (*M* = 10 mmHg) and the two combined increased ICP conditions (*M* = 80 mmHg) *t*(7) = 3.78, *p* = 0.006. There was no statistical difference in ADH secretion between the two increased ICP conditions, nonlethal condition (*M* = 65 mmHg) and lethal condition (*M* = 100 mmHg) *t*(3) = 0.42, *p* = 0.70. Findings are consistent with the proposed model. ADH secretion increases as ICP increases.

## 4 DISCUSSION

Previous studies have reported increased ADH secretion in the presence of increased ICP. However, previous studies attempted to explain increased ADH secretion within the matrix of traditional osmolarity models, that is, as plasma osmolality increases, ADH secretion increases. These studies were unable to adequately explain increased ADH secretion when plasma osmolality was low or within normal limits. Our proposed model explains both increased ADH secretion when plasma osmolality is increased and when plasma osmolality is low or within normal limits. During conditions of increased ICP, ADH secretion is independent of plasma osmolality.

When data over the past 70 years is reanalyzed within the matrix of our proposed model, all previously unexplained increase in ADH, by plasma osmolality models, can now be explained. Our study reveals this relationship is consistent and reliable. This finding is repeatedly confirmed across multiple studies, utilizing many different research study designs, for example, between groups, repeated measures within groups, single case studies, and across a broad range of subject populations, for example, adult, children, hospitalized patients, healthy individuals, and animals.

Mechanical pressure exerted on the hypothalamic nuclei results in an increased release of ADH independent of plasma osmolality. These results, independently and in combination support our hypothesis. Increased ICP stimulates specific hypothalamic nuclei, particularly paraventricular and supra‐optic nuclei, resulting in the release of ADH independent of plasma osmolality.

This is substantially different from traditional models, which rely exclusively on plasma osmolality as a stimulus for ADH release. The mechanical pressure model explains release of ADH, previously unexplained by traditional, plasma osmolality models. The proposed model has practical implications and applications for the practicing physician. For example, patients evidencing hyponatremia typically reveal high plasma osmolality. Interventions, for example water restriction, typically reduce ADH secretion and returns plasma osmolality to normal levels. However, in cases of increased ICP, water restriction often returns plasma osmolality to normal levels, while ADH continues to be released and the hyponatremia persist. Patients evidencing increased ICP, low or normal plasma osmolarity, and continuing hyponatremia can now be understood, within a new evidence based model and necessary modifications made to standard treatment protocols.

This study has limitations. Specifically, many of the studies reviewed failed to report ICP and ADH within the same article. Not all the articles reviewed provided a direct measurement of ICP, ADH in plasma, ADH in CSF, ADH in urine, or plasma osmolarity. Some studies failed to report findings in standard units, choosing instead to report findings as a percentage change, making direct comparison between studies more difficult. While our study has limitations, the data and evidence revealed are persuasive.

Where do we go from here? In order to further advance our understanding and the professional knowledge base, it will be of value, whenever possible, to measure increased ICP, plasma ADH, and plasma osmolality simultaneously. It will of particular interest to investigate and measure plasma ADH and plasma osmolality in the specific patient population evidencing hyponatremia in the presence of increased ICP and normal plasma osmolality. Collecting and reporting these measurements in combination should contribute to further understanding the relationship in ADH secretion associated with changes in ICP and contribute to the effective clinical management and treatment of patients presenting with specific electrolyte imbalances resulting from increased ICP whether the cause is brain disease or brain trauma.

## 5 CONCLUSIONS

The data extracted and reanalyzed from the professional literature, spanning the past 70 years, support the newly proposed model. ADH secretion increases in the presence of increased ICP. Mechanical pressure as the primary mechanism of action producing ADH secretion is offered as an alternative to more traditional models emphasizing dysregulation of plasma osmo‐receptors. ADH secretion can and does occur independently of plasma osmolality regulatory mechanisms. Treatment interventions based upon prevailing medical dogma and proving ineffective can now be rationally modified.

## CONFLICT OF INTERESTS

None declared.

## References

[brb31005-bib-0001] Brown, D. , & MacDonald, J. (1981). Antidiuretic hormone changes with acute increased intracranial pressure. Pediatric Research, 15, 1569 https://doi.org/10.1203/00006450-198112000-00202

[brb31005-bib-0002] Cotton, M. , Donald, P. , Schoeman, J. , Van Zyl, L. , Aalbers, C. , & Lombard, C. (1993). Raised intracranial‐pressure, the syndrome of inappropriate antidiuretic‐hormone secretion, and arginine vasopressin in tuberculous meningitis. Childs Nervous System, 9(1), 10–15. https://doi.org/10.1007/BF00301927 10.1007/BF00301927 8481936

[brb31005-bib-0003] Gaufin, L. , Skowsky, W. , & Goodman, S. (1977). Release of antidiuretic hormone during mass‐induced elevation of intracranial pressure. Journal of Neurosurgery, 46(5), 627–637. https://doi.org/10.3171/jns.1977.46.5.0627 403253 10.3171/jns.1977.46.5.0627

[brb31005-bib-0004] Gupta, G. (2015 Sept 17). Intracranial pressure monitoring. Emedicine Medscape. Retrieved from http://emedicine.medscape.com/article/1829950-overview

[brb31005-bib-0005] Mather, H. , Ang, V. , & Jenkins, J. (1981). Vasopressin in plasma and CSF of patients with subarachnoid haemorrhage. Journal of Neurology, Neurosurgery & Psychiatry, 44(3), 216–219. https://doi.org/10.1136/jnnp.44.3.216 10.1136/jnnp.44.3.216 PMC490894 7229644

[brb31005-bib-0006] Miura, M. , Takagi, S. , Matsukado, Y. , & Ushio, Y. (1989). Influence of vasopressin level on osmotic pressure and sodium concentration in plasma and cerebrospinal fluid in patients with intracranial lesions. Neurologia Medico‐Chirugica, 29, 806–810. https://doi.org/10.2176/nmc.29.806 10.2176/nmc.29.806 2480537

[brb31005-bib-0007] Moher, D. , Liberati, A. , Tetzlaff, J. , & Altman, D. (2009). The PRISMA group. Preferred reporting items for systematic reviews and meta‐analyses: The PRISMA statement. PLoS Medicine, 6(7), e1000097 https://doi.org/10.1371/journal.pmed1000097 19621072 10.1371/journal.pmed.1000097 PMC2707599

[brb31005-bib-0008] Sazbon, L. , & Groswasser, Z. (1990). Outcome in 134 patients with prolonged posttraumatic unawareness: Part 1, parameters determining late recovery of consciousness. Journal of Neurosurgery, 72(1), 75–80. https://doi.org/10.3171/jns.1990.72.1.0075 2294188 10.3171/jns.1990.72.1.0075

[brb31005-bib-0009] Shiel, R. , Pinilla, M. , & Mooney, C. (2009). Syndrome of inappropriate antidiuretic hormone secretion associated with congenital hydrocephalus in a dog. Journal of the American Animal Hospital Association, 45(5), 249–252. https://doi.org/10.5326/0450249 19723849 10.5326/0450249

[brb31005-bib-0010] Sørensen, P. , Gjerris, A. , & Hammer, M. (1985a). Cerebrospinal fluid and plasma vasopressin during short‐time induced intracranial hypertension. Acta Neurochirurgica, 77, 46–51. https://doi.org/10.1007/BF01402305 4036677 10.1007/BF01402305

[brb31005-bib-0011] Sørensen, P. , Gjerris, A. , & Hammer, M. (1985b). Cerebrospinal fluid vasopressin in neurological and psychiatric disorders. Journal of Neurology, Neurosurgery, and Psychiatry, 48(1), 50–57. https://doi.org/10.1136/jnnp.48.1.50 10.1136/jnnp.48.1.50 PMC1028182 3973621

[brb31005-bib-0012] Vingerhoets, F. , & De Tribolet, N. (1988). Hyponatremia hypo‐osmolarity in neurosurgical patients. Appropriate secretion of ADH and cerebral salt wasting syndrome. Acta Neurochirurgica, 91(1–2), 50–54. https://doi.org/10.1007/BF01400528 3394548 10.1007/BF01400528

[brb31005-bib-0013] Wians, F. (2015). Normal laboratory values. Merck manual professional edition. Retrieved from http://www.merckmanuals.com/professional/appendixes/normal-laboratory-values/normal-laboratory-values

[brb31005-bib-0014] Widmayer, M. , Browning, J. , Gopinath, S. , Robertson, C. , & Baskin, D. (2010). Increased intracranial pressure is associated with elevated cerebrospinal fluid ADH levels in closed‐head injury. Neurological Research, 32(10), 1021–1026. https://doi.org/10.1179/016164110X12714125204155 20810023 10.1179/016164110X12714125204155

[brb31005-bib-0015] Wijdicks, E. , Vermeulen, M. , Tenhaaf, J. , Hijdra, A. , Bakker, W. , & Vangijn, J. (1985). Volume depletion and natriuresis in patients with a ruptured intracranial aneurysm. Annals of Neurology, 18(2), 211–216. https://doi.org/10.1002/(ISSN)1531-8249 4037761 10.1002/ana.410180208

[brb31005-bib-0016] Wilczynski, C. (2014 May 14). Urine osmolality. Reference range. Emedicine Medscape. Retrieved from http://emedicine.medscape.com/article/2088250-overview

[brb31005-bib-0017] Wise, B. (1968). Inappropriate secretion of ADH caused by obstruction of ventriculoatrial shunts. Journal of Neurosurgery, 28(5), 429–433. https://doi.org/10.3171/jns.1968.28.5.0429 5659571 10.3171/jns.1968.28.5.0429

[brb31005-bib-0018] Yoshino, M. , Yoshimi, Y. , Taniguchi, M. , Nakamura, S. , & Ikeda, T. (1999). Syndrome of inappropriate secretion of antidiuretic hormone associated with idiopathic normal pressure hydrocephalus. Internal Medicine (Tokyo, Japan), 38(3), 290–292. https://doi.org/10.2169/internalmedicine.38.290 10.2169/internalmedicine.38.290 10337945

